# eHealth Literacy and Cyberchondria Severity Among Undergraduate Students: Mixed Methods Study

**DOI:** 10.2196/63449

**Published:** 2025-02-03

**Authors:** Wan -Chen Hsu

**Affiliations:** 1 Center for Teaching & Learning Development , National Kaohsiung University of Science and Technology, No.1, University Rd., Yanchao District, Kaohsiung, Taiwan, +886-7-3814526 ext. 31171

**Keywords:** eHealth literacy, undergraduate student, cyberchondria, compucondria, web-based health information, health information seeking, college students

## Abstract

**Background:**

With the development of the internet, health care websites have become increasingly important by enabling easy access to health information, thereby influencing the attitudes and behaviors of individuals toward health issues. However, few studies have addressed public access to health information and self-diagnosis.

**Objective:**

This study investigated the background factors and status of cyberchondria severity among college students by conducting a nationwide sample survey using the Cyberchondria Severity Scale. Further, we explored the perspective of eHealth literacy of those with scores higher than 1 SD from the mean by analyzing their recent experiences using web-based health information.

**Methods:**

A nationally representative sample of college students was surveyed, and 802 valid responses were obtained (male: 435/802, 54.2%; female: 367/802, 45.8%; mean age 20.3, SD 1.4 years). The Cyberchondria Severity Scale was used, which consisted of 4 dimensions (increased anxiety, obsessive-compulsive hypochondria, perceived controllability, and web-based physician-patient interaction). Additionally, we recruited 9 volunteers who scored more than 1 SD above the mean for in-depth interviews on their web-based health information–seeking behaviors.

**Results:**

Significant differences were found across the 4 dimensions of cyberchondria severity (F_3,2403_=256.26; *P*<.001), with perceived controllability scoring the highest (mean 2.75, SD 0.87) and obsessive-compulsive hypochondria scoring the lowest (mean 2.19, SD 0.77). Positive correlations were observed between perceived controllability, web-based physician-patient interactions, increased anxiety, and obsessive-compulsive hypochondria (*r*=0.46-0.75, *P*<.001). Regression analysis indicated that health concern significantly predicted perceived controllability (β coefficient=0.12; *P*<.05) and web-based physician-patient interaction (β coefficient=0.16; *P*<.001). Interview data revealed that students often experienced heightened anxiety (8/9, 89%) and stress (7/9, 78%) after exposure to web-based health information, highlighting the need for improved health literacy and reliable information sources.

**Conclusions:**

The study identified both benefits and risks in college students’ use of web-based health information, emphasizing the importance of critical consciousness and eHealth literacy. Future research should examine how college students move from self-awareness to actionable change and the development of critical health literacy, which are essential for effective digital health engagement.

## Introduction

eHealth information is widely accessible and essential for public health improvement. However, a digital divide persists, with individuals in rural areas facing greater barriers to internet access. The increasing demand for at-home self-diagnosis is challenged by the questionable quality of some health-related websites, where personal anecdotes and content from reputable organizations may lack proper verification, affecting users’ judgment and decision-making. Self-diagnosis websites often provide convenience but may lack oversight and include biased information, such as covert marketing for specific treatments, leading to potential exploitation [1]. This highlights the risks of relying on unverified digital health information, including misinformation, privacy issues, and harmful health decisions. eHealth literacy generally refers to an individual’s ability to obtain, read, select, critique, and use health information from the internet to solve health problems [2-4]. It comprises 3 levels: functional literacy, which refers to basic reading skills for acquiring health information; interactive literacy, involving advanced cognitive and social skills for selecting, understanding, synthesizing, and using health information in supportive environments; and critical literacy, which includes analytical and critical skills for evaluating and responding to health information, thereby improving control over life situations [5,6].

The ability to discern and ensure the trustworthiness and usability of information in a saturated web-based environment is critical for demonstrating eHealth literacy. This ability correlates with the effectiveness of individuals’ web-based health information-seeking behaviors [7]. Those with high eHealth literacy can use web-based search strategies to identify high-quality health information [8], reducing technological risks. Conversely, insufficient ability to judge web-based health information can lead to cyberchondria, which refers to excessive or repeated web searches for health-related information because of health anxiety, resulting in increased distress or anxiety [9].

Hsu [10] compared eHealth literacy between younger (18-22 years) and older (55-72 years) college students, finding significant differences in overall scores and functional literacy, with older students scoring higher. This highlights eHealth literacy’s role in helping students navigate and evaluate web-based health information, a key focus of our study. While most participants found web-based health information accessible and understandable, they were skeptical about its quality, viewing it as a mix of subjective and objective content. This underscores the importance of understanding how individuals interpret health information in a complex web-based environment.

Individuals searching for health-related information on the internet seek reassurance. However, information found on the internet is not always accurate or reliable, potentially increasing uncertainty, worry, and anxiety and leading to incorrect self-diagnoses [11,12]. Chen and Lee [13] noted that people often use limited skills to search for and evaluate vast amounts of web-based information of varying quality. However, accessing health information websites can help with self-diagnosis, and excessive concern over health may lead to cyberchondria, causing anxiety and serious health risks that arise from misinterpreting symptoms based on unverified web-based information, potentially leading to undue worry or even dangerous health actions [14]. Myrick [15] conducted a naturalistic experiment with 380 Americans to examine how emotions influence attitudes and behaviors following an information search. The study identified several emotions—fear, hope, satisfaction, interest, and motivation—that impact individuals’ reactions to web-based health information. In particular, Myrick highlighted that social cognitive factors, such as self-efficacy and outcome expectations, positively influence confidence and behavioral intentions when searching for health information. This finding emphasizes how emotions and cognitive assessments together encourage or deter health-related actions, demonstrating the significant role of social cognitive factors in web-based health information–seeking behaviors.

Further, Starcevic and Berle [9] highlighted that when individuals with high health anxiety search for health information on the internet, receiving timely reassurance from others (particularly doctors) can alleviate their anxiety and reinforce their behavior of searching for health information. Conversely, if the search process increases anxiety or panic, users may continue searching to alleviate their negative emotions. However, repeated disappointment can worsen their emotional burden, deepen cyberchondria severity, and potentially lead to the avoidance of web-based health information searches in the future.

Providing web-based health information and ensuring public access are significant technological issues in society today. However, the “prescriptions” found on the internet—advice or treatments from articles or user testimonials without professional oversight—often offer only temporary relief, potentially causing anxiety and worry. These web-based prescriptions can mislead individuals, resulting in inappropriate or ineffective self-treatment, underscoring the importance of reliable, professionally vetted health information to support safer health decision-making. Additionally, the difficulty of verifying the credibility of web-based health information often leads users to rely on self-diagnosis based on their symptoms, which can increase health-related anxiety. This is particularly concerning when individuals lack the tools to assess the reliability of web-based sources, potentially resulting in misinformation and heightened worry. Adolescents, for instance, frequently report feeling overwhelmed by ambiguous health data on the internet, sometimes exacerbating their fears about symptoms they may not fully understand [16].

Most people searching for web-based health information lack medical training. Specifically, being exposed to a vast amount of web-based health information often causes anxiety and emotional distress because of the inability to understand medical terms or judge the information, resulting in severe self-diagnosis reactions [17]. Shan et al [18] found that adults generally have low levels of critical health literacy. Developing this literacy through education can enhance the ability to critically assess health information, engage in critical dialogues, and make health-related decisions.

Further, the measurement methods for cyberchondria severity are in developmental stages. Early studies have measured cyberchondria using a single item; however, later studies have developed multidimensional and multi-item scales. These scales include dimensions such as compulsion (excessive searching for web-based health information), distress (negative emotions and physiological responses from searching), reassurance (seeking reassurance from medical professionals), and mistrust of medical professionals (conflict between trusting web-based health information and doctors) [19]. Starcevic [20] considered cyberchondria to be a psychological construct. Hsu et al [3] found that college students with high health standards and high health concerns develop better eHealth literacy and adopt healthy eating, exercising, and better sleeping behaviors. Thus, in addition to developing reliable assessment tools, specific contextual items for different populations should be considered. Therefore, this study includes health status and health concerns to explore their impact on cyberchondria.

Specifically, this study aims to conduct a national survey using the Cyberchondria Severity Scale to understand the background factors and current status of cyberchondria among college students; recruit participants whose Cyberchondria Severity Scale scores are more than 1 SD above the mean and explore their experiences using web-based health information from the perspective of eHealth literacy; and based on the phased research results, summarize the characteristics of college students’ experiences with web-based health information using the eHealth literacy framework and provide specific recommendations for developing health-promotion competencies to enhance the application value for health education practices.

## Methods

### Overview

This study used a sequential mixed methods design, which used qualitative and quantitative research sequentially, depending on the purpose or problem of the study. The purpose was to attain complementarity, such as the rationality of quantitative data in additional sampling and to investigate further through qualitative research [21]. Accordingly, this study was conducted in two phases. The first phase, comprising the quantitative part of the study, aimed to screen the respondents. The second phase, comprising interviews, aimed to collect the respondents’ eHealth literacy data, which were analyzed qualitatively.

### Recruitment

In this study, we specifically focused on Taiwanese college students. For the purposes of this research, a college student was defined as an individual aged 18‐22 years currently enrolled in an undergraduate program at a Taiwanese higher education institution. This age range aligned with the typical age group of undergraduate students in Taiwan and provided a consistent basis for analysis within this demographic. The survey was conducted in two waves: at the end of the first (September 2021) and second (June 2022) semesters. We recruited 802 participants, including students from 14 universities in Taiwan, and we administered a questionnaire to them. Subsequently, we selected 9 volunteers for interviews based on their Cyberchondria Severity Scale scores, specifically those whose scores exceeded 1 SD above the mean, indicating a higher level of cyberchondria. Initial contact was made through the survey platform, and we followed strict ethical guidelines to protect participants’ privacy throughout the recruitment and interview process.

### Ethical Considerations

Ethical approval was granted by the Ethics Research Committee of National Cheng Kung University (No. 110-573-2), and informed consent was obtained from all participants. Participants had the right to withdraw from the study at any time. To preserve anonymity in the initial survey, all responses were collected without identifiable information. For follow-up interviews, we requested separate, voluntary consent from participants who met the selection criteria. All methods were carried out per relevant ethical standards, guidelines, and regulations. Participants who completed the initial survey received a US $3 gift voucher as a token of appreciation for their time and effort. Additionally, participants who took part in the follow-up interviews were compensated with a US $15 gift voucher to acknowledge their contribution to the study. These measures aimed to encourage participation while ensuring voluntary involvement without undue influence. By implementing these compensation protocols, we maintained ethical practices that aligned with institutional guidelines and ensured the rights and welfare of the participants were upheld throughout the research process.

### Survey

The Cyberchondria Severity Scale was modified from those proposed by Durak Batigun et al [22] and White and Horvitz [23]. A 5-point Likert scale was used, with the following responses: (1) never, (2) rarely, (3) sometimes, (4) often, and (5) always. The exploratory factor analysis led to a Cyberchondria Severity Scale for college students with 4 constructs: increased anxiety (Cronbach *α*=0.91), obsessive-compulsive hypochondria (Cronbach *α*=0.87), perceived controllability (Cronbach *α*=0.88), and web-based physician-patient interaction (Cronbach *α*=0.86). The total scale Cronbach α was 0.92, with a variance of 66.81%. The confirmatory factor analysis indicated that item reliability ranged from 0.50 to 0.86, factor loadings ranged from 0.71 to 0.93, and the composite reliability for latent variables ranged from 0.83 to 0.90 (*P*<.001). The extracted average variance was from 0.46 to 0.60. Increased anxiety referred to the anxiety experienced by an individual due to apprehensions about web-based content-seeking to understand a disease or its symptoms (eg, “To understand a certain disease or symptom, I went on the internet to search for information. The large amount of information was too much for me to handle and increased my anxiety”). Obsessive-compulsive hypochondria referred to excessive worry or excessive internet surfing to understand a disease or its symptoms (eg, “I feel that I am someone who worries excessively about health”). Perceived controllability referred to the decreased doubts or psychological burdens caused by a web search for health information (eg, “Searching for health-related information on the internet allows me to understand my medical condition and decreases my anxiety”). Web-based physician-patient interaction refered to an individual’s web-based information search that affected their judgment of their symptoms (eg, “After seeing a physician, I would use the internet to obtain detailed information on the diagnosis”).

To understand the advantages, disadvantages, and risks of college students’ access to web-based health information, this study used semistructured interviews. The interview outline was developed based on the research objectives and focused on collecting information about the background of frequent web-based health information users who engaged in self-diagnosis, as well as their experiences, including the advantages, disadvantages, and risks. This information was gathered from college students.

### Data Analysis

In the first phase of the study, SPSS version 20 (IBM Corp) was used to calculate the mean, SD, and dependent‐sample one-way ANOVA to indicate the current status and differences across various dimensions of college students’ cyberchondria. In the second phase, the Strauss and Corbin constant comparative method was used to analyze the advantages, disadvantages, and risks of web-based health information [23]. Open coding was first conducted by assigning codes and noting data entry dates. For example, for Participant 8-20220920, the number 20220920 indicates that the interview with Participant 8 was conducted on September 20, 2022. Thereafter, the researcher read the transcripts, conceptualized the data, and grouped similar concepts into categories. Finally, axial coding was conducted to link the subcategories and main categories. This study followed Lincoln and Guba’s [24] credibility, dependability, and confirmability criteria to ensure research quality. With the participants’ consent, data were collected by recording and note-taking to ensure completeness and accuracy.

## Results

### Current Situation of Cyberchondria Severity Among College Students

The sociodemographic details of the sample are shown in Table 1. Regarding the different dimensions of cyberchondria, the average single-item scores ranged from 2.19 to 2.75 (between rarely and sometimes). The average scores for each dimension were below the median, indicating that college students did not frequently experience hypochondria during web-based health information searches. However, they did not often feel that their anxiety was controllable or engage in web-based physician-patient interactions (Table 2).

**Table 1.** Sociodemographic details of the sample (n=802).

Variable and group	Frequency, n (%)
Sex	
Male	435 (54.2)
Female	367 (45.8)
Grade	
Freshman year	565 (70.4)
Sophomore year	74 (9.2)
Junior year	87 (10.8)
Senior year	76 (9.5)
Health status	
Very unhealthy	4 (5)
Unhealthy	53 (6.6)
Neutral	252 (31.4)
Healthy	298 (37.2)
Very healthy	195 (24.3)
Degree of health concern	
Very unconcerned	12 (1.5)
Unconcerned	55 (6.9)
Neutral	266 (33.2)
Concerned	287 (35.8)
Very concerned	182 (22.7)

**Table 2.** Descriptive statistics of cyberchondria severity (n=802).

Single-item	Minimum score	Maximum score	Likert score, mean (SD)
Increased anxiety	1	5	2.39 (0.82)
Obsessive-compulsive hypochondria	1	5	2.19 (0.77)
Perceived controllability	1	5	2.75 (0.87)
Online physician–patient interaction	1	5	2.70 (0.80)

Owing to the different number of items in the 4 dimensions of cyberchondria among college students, a dependent‐sample one-way ANOVA was conducted using the single-item mean scores of the 4 dimensions. The results showed significant differences across the dimensions (F_3,2403_=256.26; *P*<.001), indicating significant variations in cyberchondria scores among college students across different dimensions. Further post hoc comparisons using the least significant difference test revealed that perceived controllability had the highest score, with the highest single item being “When I search for health-related information on the internet, reading about my condition from reliable sources (eg, hospital websites) alleviates my anxiety” (mean 2.79, SD 0.75). Obsessive-compulsive hypochondria scored the lowest, indicating that college students did not consider themselves to be overly concerned about their health (Table 3).

**Table 3.** Dependent‐sample one-way ANOVA of the different dimensions of cyberchondria severity among college students.

Analysis	Type III sum of squares (*df*)	Mean square
Between groups	513.11 (801)	0.64
Within groups	513.11 (801)	0.64
Each dimension^a^	513.61 (3)	171.20
Residual error	1605.41 (2403)	0.668
Total	2632.13 (3207)	—

a Post hoc test revealed perceived controllability>web-based physician–patient interaction>increased anxiety>obsessive-compulsive hypochondria (*F*_3,2403_=256.26, *P*<.001).

The correlation matrix of different dimensions indicated significant positive correlations between perceived controllability, web-based physician-patient interaction, increased anxiety, and obsessive-compulsive hypochondria. This suggests that the more college students feel they can control their anxiety by searching for web-based health information and the more frequently they interact with web-based physicians, the higher their levels of anxiety and obsessive-compulsive hypochondria. Further, perceived controllability exhibited a significant positive correlation with web-based physician-patient interactions. The absolute values of the correlation coefficients were all above 0.40, indicating moderate-to-high correlations. Thus, the most significant dimensions of cyberchondria among college students showed moderate or high correlations (Table 4).

**Table 4.** Correlation analysis of different dimensions of cyberchondria severity among college students.

Variable	Increased anxiety	Obsessive-compulsive hypochondria	Perceived controllability	Online physician–patient interaction
Increased anxiety				
*r*	1.00	0.75	0.46	0.48
*P* value	—^a^	<.001	<.001	<.001
Obsessive-compulsive hypochondria				
*r*	0.75	1.00	0.50	0.54
*P* value	<.001	—	<.001	<.001
Perceived controllability				
*r*	0.46	0.50	1.00	0.73
*P* value	<.001	<.001	—	<.001
Online physician–patient interaction				
*r*	0.48	0.54	0.73	1.00
*P* value	<.001	<.001	<.001	—

a Statistical analysis was not done for reference values.

Table 5 indicates that all individual variables were significant predictors of the 4 dimensions of cyberchondria severity, with an overall explanatory power of 1%‐2%, corresponding with a low predictive power level. Notably, the degree of health concern emerged as a significant positive indicator of all dimensions of cyberchondria severity, except increased anxiety.

**Table 5.** Multiple regression analyses of individual factors predicting cyberchondria severity.

Variable	Increased anxiety^a^				Obsessive-compulsive hypochondria^b^				Perceived controllability^c^				Web-based physician-patient interaction^d^			
	B^e^	β^f^	*t* test (*df*)	*P* value	B	β	*t* test (*df*)	*P* value	B	β	*t* test (*df*)	*P* value	B	β	*t* test (*df*)	*P* value
Health status	−0.09	−0.10	−2.32 (790)	.02	−0.11	−0.13	−2.87 (790)	.006	0.00	0.00	0.07 (790)	.43	−0.01	−0.01	−0.29 (790)	.06
Degree of health concern	0.01	0.01	0.18 (790)	.08	0.11	0.13	2.93 (790)	.004	0.11	0.12	2.58 (790)	.02	0.14	0.16	3.69 (790)	<.001

a *R*=0.10, *R*2=0.01 (*P*=.02); *F*_3,790_=2.73 (*P*=.02).

b *R*=0.12, *R*2=0.01 (*P*=.02); *F*_3,790_=3.55 (*P*=.02).

c *R*=0.12, *R*2=0.02 (*P*=.002); *F*_3,790_=3.90 (*P*=.003).

d *R*=0.16, *R*2=0.02 (*P*<.001); *F*_3,790_=6.54 (*P*<.001).

e B: unstandardized coefficients.

f β: standardized coefficients.

### Impact of Accessing Web-Based Health Information on the Physical and Mental Well-Being of College Students

The interviews with college students revealed that accessing web-based health information has both psychological and physical effects, often creating challenges due to the nature and quality of the information available on the internet. In terms of psychological impact, participants reported feeling overwhelmed by the plethora of opinions and conflicting advice, which often caused confusion and heightened anxiety. For example, one student shared the following:

*I could not find any results through searching, so I mainly see a doctor because I have no other option. I don’t know what to do anymore, so I must see a doctor. And since I’ve been paying more attention to health issues, I’ve noticed that the first things that pop up are usually some exaggerated news or bizarre articles from content farms. The headlines make me feel like, “Oh my god! I'm about to die*.*”*

[Participant 8-20220920]

This highlights the stress caused by exaggerated or unreliable health information, which drives the students to consult health care professionals as a last resort. As for strained doctor-patient relationships, the tendency to self-diagnose based on web-based information sometimes led students to question or dismiss professional medical advice, creating tension in doctor-patient interactions. One student remarked the following:

*If you have doubts about the doctor’s words, don’t ask random questions. For instance, if I now Google some issues, like searching for stomach pain on Google, and then tell the doctor, “But Google says this is some specific disease, so you just need to prescribe me this medicine,” it can lead to problems*.

[Participant 1-20221101]

This underscores the potential of web-based health information to undermine the trust in medical expertise. Additionally, exposure to sensational or clickbait content exacerbated feelings of fear and helplessness. Students described how alarming headlines led to unnecessary worry:

*And since I’ve been paying more attention to health issues, I’ve noticed that the first things that pop up are usually some exaggerated news… The headlines make me feel like, “ Oh my god! I'm about to die*.*”*

[Participant 8-20220920]

The physical impact included misguided health practices and delayed medical treatment. Some students engaged in health practices based on unverified advice on the internet, which sometimes led to negative outcomes. For example, one participant shared a personal experience:

*My chest is not very big, and I saw online that eating green papaya can make your chest bigger. I was just in middle school then, so I started eating one every day… Then I noticed that my hands were starting to turn yellow.*

[Participant 9-20221024]

Moreover, in some cases, students delayed seeking professional care due to reliance on web-based advice. One participant shared a story about their friend:

*Like my friend, he once sprained his finger while playing basketball… He looked it up online and found advice saying to ice it and not press it. He kept doing that, and now his finger is crooked.*

[Participant 3-20221107]

This highlights the risk of self-experimentation based on health misinformation and demonstrates how misleading information can lead to improper self-care and long-term consequences. The findings emphasize the dual nature of web-based health information. Although such information is accessible and convenient, its unreliable or sensational nature can lead to considerable psychological and physical challenges for students. This emphasizes the urgent need for improving students’ health literacy on the internet and promoting access to credible and professionally vetted health information.

### College Students’ Experiences With Accessing Web-Based Health Information: The Advantages, Disadvantages, and Risks

Students’ responses to the semistructured interviews revealed convenience, richness of information, privacy, and validation as the 4 main themes related to using web-based health information. The motivation for using web-based health information was to meet the needs of beautifying features, weight loss, and fitness. The two types of web-based health information access were fixed webpage browsing and nonfixed webpage retrieving. The criteria for checking the quality of web-based health information included subjective judgments and objective features. Regarding web-based health information reading experiences, problems were caused by technical terms, foreign languages, and numerical health information data. Typically, the participants searched for related information on the internet to clarify their doubts. Finally, the impacts of using web-based health information depend on assessing its quality, lifestyle changes, and the possibility of self-practice. Self-diagnosis can be a double-edged sword. It may empower individuals with preliminary health insights; however, it also poses risks, particularly if the information is misinterpreted or lacks professional verification. Individuals should be encouraged to evaluate web-based health information and approach self-diagnosis with caution. Additionally, we will consider the ethical implications of promoting self-diagnosis in health-related research, emphasizing the importance of fostering critical eHealth literacy among users as a suggestion.

The participants reported various risks and benefits while searching for health information on the internet. They tended to be wary of sensational or emphatically presented headlines, leading them to adopt a conservative approach to avoid harm. In assessing the quality of information, they paid attention to the source, crosschecked it with multiple sources, and judged its effectiveness based on their personal experiences and expert endorsements, ensuring content completeness. Their experiences included selective understanding, focusing on general ideas rather than technical terms, and further clarifying doubtful information. The impact of this information varied based on the closeness of relationships with others and the feasibility of implementing lifestyle changes. For instance, support from close friends and family could enhance the likelihood of adopting positive health behaviors and reduce anxiety related to conflicting information. Conversely, a lack of support or conflicting opinions from peers could heighten stress and uncertainty, making it more challenging to discern and apply accurate health information.

Summarizing the experiences of college students who engaged in self-diagnosis through extensive internet use for health information access, the benefits primarily included cross-referencing, synthesizing diverse information, and verifying questionable information. The harms mainly arose from confusion due to difficulty reading and understanding the diverse information and, in more severe cases, engaging in self-experimentation that affected physical health. The risks involved selective understanding, blind sharing of information, and extreme rejection of any information, which could undermine the positive intentions of effectively using web-based health information. Table 6 presents the interview analysis and coding framework.

**Table 6.** Interview analysis and coding framework.

Main and subcategory structure	Interviewee numbers
Background of using web-based health information	
Time of initial use	
I mostly started in high school	1, 3, 4, 6, 7, 8, 9
Initial motivation for using web-based health information	
Knowledge acquisition	1, 2, 4, 5, 6, 8, 9
Concern for one’s condition	1, 2, 3, 4, 5, 6, 7, 8, 9
Searching for health and medical information for family	2, 5, 6, 8, 9
Searching for drug ingredients	2, 3, 5, 6, 7, 8
Looking for nearby medical facilities	1, 2, 4, 7, 8
Reason for choosing web-based health information	
Free of charge	1, 2, 3, 4, 6, 8
Richness of information	1, 2, 5, 7, 8, 9
Verifiability	1, 2, 3, 4, 5, 6, 7, 8
Convenience	1, 2, 4, 5, 7, 8, 9
Experience in accessing web-based health information	
Quality assessment of web-based health information: subjective judgment	
Sensational headlines	1, 4, 5, 6, 8
Headlines overly emphasizing efficacy	1, 4, 5, 6, 7, 8, 9
Conservative adoption to avoid harm	2, 3, 5, 7, 8
Judged by my common sense	1, 3, 5, 6, 8, 9
Quality assessment of web-based health information: objective standards	
Source of information	1, 2, 4, 5, 6, 8
Cross-verification	2, 3, 5, 6, 7, 8, 9
Judged by actual experiences of friends and family	1, 2, 3, 4, 7, 8
Expert evaluation of the efficacy of information	1, 3, 4, 5, 6, 7
Incomplete information	2, 3, 4, 5, 6, 9
Experience in reading web-based health information	
Summary understanding and further inquiry	
Handling doubts about web-based health information: continue searching or ask others interesting questions	1, 2, 3, 4, 5, 6, 7, 8, 9
Understanding of web-based health information: often ignoring technical terms and focusing on general meaning	1, 4, 5, 6, 8, 9
Impact of web-based health information	
Deliberation on object and risk outcome assessment	
Influence on others based on personal relationships: varying forms and degrees of influence	1, 2, 3, 4, 7, 9
Self-physical and mental impact: depending on the extent of lifestyle changes or feasibility	1, 2, 3, 4, 5, 6, 7, 8, 9

## Discussion

### Principal Findings

Digital platforms have revolutionized health and science by enabling collaboration, expanding evidence-based interventions, and advancing public health. Early successes in behavioral medicine demonstrated their potential to scale therapeutic solutions. With growing investments in health information technology, behavioral informatics has become essential, integrating medicine, psychology, and engineering to develop patient apps, clinical tools, communication programs, and population health strategies. The Society of Behavioral Medicine, positioned at this interdisciplinary nexus, plays a pivotal role in shaping how science is applied to reduce health disparities, support patients and caregivers, enhance community well-being, and address challenges such as psychological pain and addiction [25]. This study examined eHealth literacy and cyberchondria severity in college students, highlighting their web-based health information-seeking behaviors. Key findings include links between perceived controllability, web-based interactions, anxiety, and obsessive-compulsive hypochondria, with health concern predicting controllability and interactions. Interviews showed that 89% (8/9) felt heightened anxiety, and 78% (7/9) reported stress from web-based health information.

These results highlight the dual nature of web-based health information. The high perceived controllability scores suggest that accessing reliable web-based health information—such as from trusted sources like hospital websites—can alleviate student anxiety by helping them manage health-related stress. This finding aligns with prior research showing the importance of credible sources in fostering a sense of control [10,23]. By contrast, the low obsessive-compulsive hypochondria scores suggest that students generally do not exhibit excessive worry or compulsive searching behaviors. This is contrary to the studies linking frequent web-based health searches to increased distress and cyberchondria severity [9]. This discrepancy may stem from differences in digital literacy or cultural attitudes toward web-based health information.

The positive association between health concern and cyberchondria dimensions underscores the dual impact of web-based health information. Although such information can promote awareness and self-management, it can also lead to anxiety and stress when students encounter conflicting or sensational content; this is consistent with the findings from Tan and Goonawardene’s systematic review [26]. Moreover, the role of web-based physician-patient interactions in moderating anxiety highlights the importance of guiding students toward appropriate and reliable eHealth resources. This aligns with Hsu’s [10] findings that enhancing eHealth literacy can empower students to critically evaluate web-based health information and reduce health-related anxiety.

Notably, the interviews revealed that excessive web-based health searches contribute to confusion and emotional distress, particularly when students encounter contradictory advice or exaggerated claims. These results align with those of Starcevic and Berle [9], who identified increased anxiety and obsessive-compulsive tendencies as key outcomes of frequent health information searches. Furthermore, exposure to sensational content, such as clickbait headlines, exacerbates fear and helplessness, increasing the likelihood of unnecessary medical consultations or misdiagnoses [11,23]. These findings emphasize the need to develop interventions that improve students’ critical health literacy, which would enable them to discern credible information and mitigate the psychological impacts of misinformation.

This study has limitations. Self-reported data may introduce response bias, suggesting the need for objective health measures like medical records for greater reliability. The qualitative phase included only 9 participants, restricting the generalizability of the findings; a larger sample could offer deeper insights. Additionally, focusing solely on Taiwanese college students limits the applicability of the results to other cultures or demographics. Cross-cultural studies are recommended to examine variations in eHealth literacy and cyberchondria across populations.

This study highlights the complex interplay between eHealth literacy and cyberchondria severity among college students, revealing both benefits and risks associated with web-based health information-seeking behavior. Reliable web-based information can reduce anxiety and enhance self-management, whereas the prevalence of contradictory or sensational content underscores the importance of fostering critical eHealth literacy. Drawing on Nutbeam’s framework of health literacy [18,27], this study advocates for integrating critical health literacy into health education programs to empower students to effectively navigate the digital health landscape.

Moreover, the findings contribute to the broader discourse on digital health education, emphasizing the need for educational interventions that promote self-awareness and actionable behavioral changes. As previous studies have shown, developing critical eHealth literacy not only improves individual decision-making but also strengthens patient-physician relationships by encouraging open and informed discussions [26,28]. Future efforts should focus on designing tailored health literacy initiatives that equip students with the skills to critically assess and apply web-based health information, fostering their ability to engage responsibly with digital health resources in a rapidly evolving technological landscape.

### Conclusions

This study surveyed college students using the Cyberchondria Severity Scale and interviewed high scorers to explore their browsing behaviors and experiences with web-based health information. Significant score differences were observed across cyberchondria dimensions, with perceived controllability scoring highest and obsessive-compulsive hypochondria lowest. High scorers noted benefits such as cross-verification and clarification of information; drawbacks like confusion from complexity; and risks including selective understanding, reflexive sharing, and mistrust, which could undermine the advantages of web-based health resources.

The internet has revolutionized medical education and practice by enabling the rapid, large-scale exchange of information and ideas across geographically dispersed audiences [29]. To effectively use web-based health information, critical awareness and eHealth literacy are essential skills. Future research should investigate how college students move from self-awareness to a behavioral change that helps them develop critical health literacy as a crucial component of digital health education.

This study relied on self-reported questionnaires, which may introduce response bias and affect the accuracy of findings, particularly regarding health status. Future studies could improve data reliability by encouraging participants to reference recent health examination results, enhancing internal validity. Additionally, the small interview sample limits the generalizability of conclusions. Expanding the sample in future research could reduce bias and support more robust findings.
